# Erythema nodosum: Atypical presentation of common disease

**DOI:** 10.4103/0970-2113.68319

**Published:** 2010

**Authors:** Jagdeep Whig, Vineet Mahajan, Anil Kashyap, Sushil Gupta

**Affiliations:** *Department of Pulmonary and Critical Care, Dayanand Medical College and Hospital, Ludhiana, Punjab, India*; 1 *Department of Critical Care, Dayanand Medical College and Hospital, Ludhiana, Punjab, India*

**Keywords:** Erythema nodosum, tuberculosis, cutaneous tuberculosis

## Abstract

Tuberculosis is a very common disease often presenting in an uncommon form. High level of suspicion is required to diagnose it, thereby preventing its morbidity and mortality. We present a case of young female with multiple tuberculo-protein hypersensitivity reactions without any evidence of active tuberculosis in the body.

## INTRODUCTION

Hypersensitivity reaction to tuberculo-protein can be the first and only presentation of tuberculosis in a patient with / without evidence of active disease. These reactions can be of various types like phlyctenular conjunctivitis, erythema nodosum, increased dermal protein hypersensitivity, pleural or pericardial effusion and reactive polyarthritis. But simultaneous occurence of more than one reaction in a single patient without any evidence of primary disease has been reported very infrequently.

## CASE REPORT

A 32 year old female was admitted in our department with complaints of multiple painful skin nodules over both legs with off and on pain in both knee and ankle joints from last two months. There were no symptoms of cough, expectoration, fever, weight loss or any gynecological complaint. She was non diabetic with no history of any contact of tuberculosis in the family. There was no history of similar episode or any drug rash in the past. She did not take any medication, including oral contraceptive pills, before the onset of skin rash. She did not respond to antibiotic (amoxicillin + clavulanic acid) and pain killers prescribed by a private practitioner.

**Figure 1:**
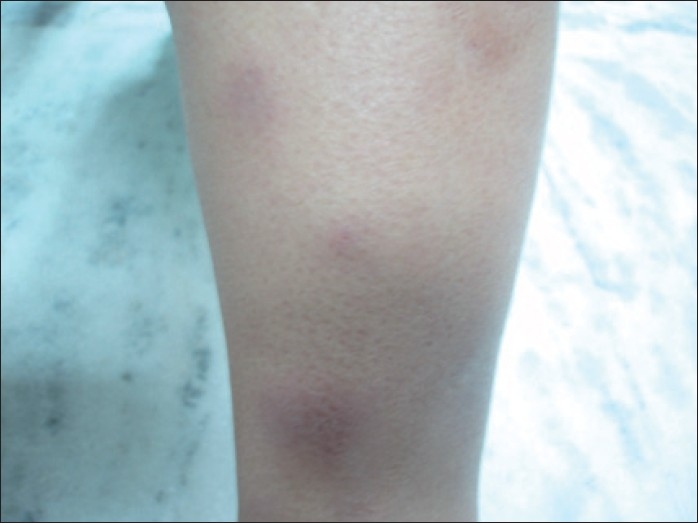
Erythematous, papulo-nodular skin lesions of 8-10 mm size over the shin

On general physical examination, she was pale with multiple erythematous, tender, papulo-nodular skin lesions of 8 - 10 mm size over both legs, more on the shins, compatible with erythema nodosum [Figure 1]. The knee and ankle joints of both legs were reddish and tender without any obvious swelling. Her resting pulse rate was 88 / min, blood pressure was 110/72 mm Hg and respiratory rate was 22 / min. Respiratory, cardiovascular, abdominal and central nervous system examination were within normal limits. Routine blood investigations were all normal except erythrocyte sedimentation rate, which was 114 mm 1^st^ hr Westergren. Her mantoux test was strongly positive with 25 mm induration with vesicle formation over the site. Chest radiograph was within normal limits

**Figure 2:**
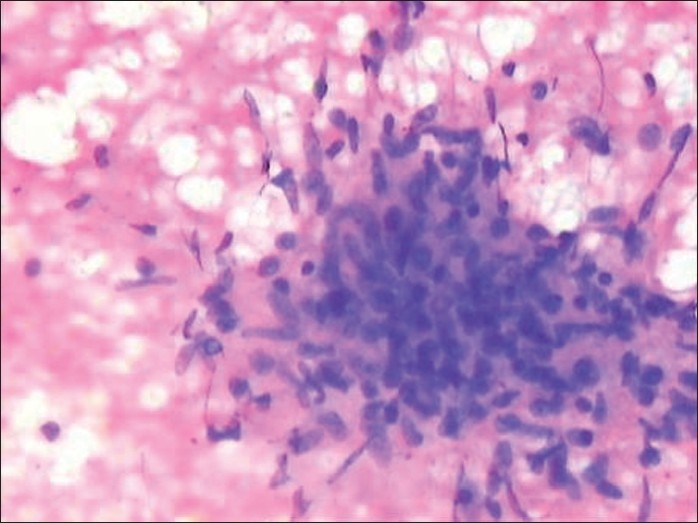
Histopathology showing multiple epitheloid cell granulomas with Langhans giant cell reaction in subcutaneous tissue without any evidence of caseous necrosis

Radiographs of bilateral ankle and knee joints were also normal. Ultrasound abdomen was normal without any evidence of lymphadenopathy. Normal Anti Streptococcal O (ASO) titer ruled out any rheumatic process in the body. Rheumatoid Factor (RA), Anti Nuclear Antibody (ANA) and C - Reactive Protein (CRP) were negative. Skin biopsy was done from the right shin and the mantoux site. Histopathological examination showed multiple epitheloid cell granulomas with Langhans giant cell reaction in subcutaneous tissue without any evidence of caseous necrosis [Figure 2]. Ziehl Neelson (ZN) staining for acid fast bacilli was negative. So, a diagnosis of erythema nodosum was made and the underlying etiology was proved to be tuberculosis. She was started on category III under DOTS. Within three weeks of starting therapy, there was significant improvement in joint pains and the skin lesions resolved.

## DISCUSSION

Erythema nodosum (EN) is the most frequent acute hypodermatitis. It is an acute, nodular, erythematous eruption that is usually limited to the extensor aspects of the lower legs, with occasional spread to thighs or arms.[1] It is presumed to be a hypersensitivity reaction and may occur in association with several systemic diseases like sarcoidosis, inflammatory bowel disease, infections or drug therapies, or it may be idiopathic in 70% of cases.[2] It is the most common panniculitis in India. In a 15-year descriptive study of cutaneous tuberculosis, out of 55 cases, 48% were true cutaneous tuberculosis and 52% were tuberculids out of which erythema nodosum was the most frequent form.[3] Erythema nodosum may occur in children and patients older than 70 years, but it is more common in young adults aged 18–34 years. As a manifestation of tuberculosis, erythema nodosum has been described in children with primary tuberculosis infection and may be associated with phlyctenular conjunctivitis.[4] Low grade fever and swelling of the ankle joints accompany the skin lesions in some patients. Immune complex deposition within dermal vessels is an important component in the production of the symptom complex. Patients usually present with skin rash in the form of dusky red, tender and nodular lesions. Lesion borders are poorly defined and size vary from 2–6 cm. Individual lesions last approximately two weeks, but occasionally, new lesions continue to appear for three to six weeks. Arthralgia occurs in more than 50% of patients and begins during the eruptive phase or precedes the eruption by 2–4 weeks. Any joint may be involved, but the ankles, knees, and wrists are affected most commonly. No destructive joint changes occur. Synovial fluid is acellular, and the rheumatoid factor is negative. When erythema nodosum is diagnosed, it is important to find out the underlying conditions. These include a detailed history, including drug and oral contraceptive use, a careful physical examination and a chest X-ray. The tuberculin skin test is always strongly positive and a negative skin test rules out tuberculosis as the etiology.[5] Routine blood investigations are done to rule out any systemic infection and Computed Tomography scan is done to diagnose sarcoidosis.

Erythema nodosum tends to disappear by itself and often does not need any specific treatment. Non steroidal anti-inflammatory drugs (NSAIDS), bed rest and cool wet compresses are advised for relief of pain.[6] If the underlying cause is found, it should be treated first. Corticosteroids are needed in some cases provided that there is no infection or cancer that has acted as trigger. Generally, the prognosis of erythema nodosum is very good and most people do not have further problems.
